# Efficacy and safety of adjuvant transarterial chemoembolization in high-risk HCC after curative hepatectomy: a phase III RCT protocol

**DOI:** 10.1016/j.iliver.2026.100236

**Published:** 2026-04-25

**Authors:** Chao-Man Huang, Shu-Chang Chen, Yi-He Yan, Chuang Qin, Shu-Qun Li, Ze Su, Xiao-Feng Dong, Teng-Meng Zhong, Fan-Jian Zeng, Hong-Bing Yao, Shao-Ping Liu, Pei-Sheng Wu, Ning Peng, Jie Liu, Liang Ma, Jian-Hong Zhong

**Affiliations:** a Department of Hepatobiliary Surgery, Guangxi Medical University Cancer Hospital, Nanning 530021, China; b Department of Hepatobiliary Surgery, Wuzhou People's Hospital, Wuzhou 543001, China; c Department of General Surgery, Second Affiliated Hospital of Guangxi Medical University, Nanning 530007, China; d Department of Hepatobiliary Surgery, Liuzhou People's Hospital, Liuzhou 545006, China; e Department of Hepatobiliary Surgery, Affiliated Hospital of Guilin Medical University, Guilin 541002, China; f Department of Hepatobiliary Pancreatic Surgery, First People's Hospital of Nanning, Nanning 530016, China; g Department of Hepatobiliary, Pancreatic and Spleen Surgery, People's Hospital of Guangxi Zhuang Autonomous Region, Guangxi Academy of Medical Sciences, Nanning 530021, China; h Department of Hepatobiliary Surgery, Baise People's Hospital, Baise 533000, China; i Department of Hepatobiliary Surgery, Wuzhou Red Cross Hospital, Wuzhou 543002, China; j Department of Hepatobiliary and Pancreatic Surgery, Second Affiliated Hospital of Guilin Medical University, Guilin 541199, China; k Department of Hepatobiliary and Pancreatic Surgery, Eighth Affiliated Hospital of the Guangxi Medical University, Guigang 537100, China; l Department of Hepatobiliary and Pancreatic Surgery, First People's Hospital of Qinzhou, Qinzhou 535000, China; m Department of Hepatological Surgery, First Affiliated Hospital of Guangxi Medical University, Nanning 530021, China; n Department of Hepatobiliary Pancreatic Surgery, Guilin People's Hospital, Guilin 542001, China

**Keywords:** Adjuvant, Hepatocellular carcinoma, High-risk recurrence factors, Transarterial chemoembolization

## Abstract

**Introduction:**

Hepatocellular carcinoma (HCC) remains associated with a high risk of recurrence, with 5-year recurrence rates approaching 70% after curative hepatectomy. The Chinese Liver Cancer Staging guideline strongly recommend transarterial chemoembolization (TACE) as an adjuvant therapy to reduce postoperative recurrence and improve overall survival. However, its clinical benefit remains controversial, as guidelines from other regions do not support routine use of adjuvant TACE. This discrepancy may reflect the possibility that adjuvant TACE primarily facilitates detection of intrahepatic residual lesions through digital subtraction angiography (DSA), rather than conferring direct therapeutic benefit from embolic or chemotherapeutic agents.

**Methods and analysis:**

This multicenter, phase III randomized controlled trial will enroll 442 eligible participants, who will be randomly assigned in a 1:1 ratio to either an adjuvant TACE group or an intensive follow-up group. The primary endpoint of this study is to investigate whether adjuvant TACE could improve recurrence-free survival (RFS) in high-risk HCC patients following curative hepatectomy. The key secondary endpoints include the safety and tolerability of adjuvant TACE, as well as the effects of adjuvant TACE versus intensive follow-up on overall survival, median RFS, and time to tumor recurrence. This trial may addresses the critical clinical controversy regarding adjuvant TACE by excluding patients with residual tumor staining on DSA, to ensure genuine curative resection. The results will deliver high-quality evidence to clarify the efficacy and safety of adjuvant TACE, and standardize postoperative adjuvant strategies.

## Abbreviations

AEs: Adverse events
CNLC: Chinese Liver Cancer Staging Guidelines; CRFs, Case report forms
CT: Computed tomography; DSA, Digital subtraction angiography
ECG: Electrocardiogram
ECOG PS: Eastern Cooperative Oncology Group performance status
GCP: Good Clinical Practice
HBV: Hepatitis B virus
HCC: Hepatocellular carcinoma
HCV: Hepatitis C virus
MRI: Magnetic resonance imaging
OS: Overall survival
RCT: Randomized controlled trial
RECIST: Response Evaluation Criteria in Solid Tumors
RFS: Recurrence-free survival
SAEs: Serious adverse events
TACE: Transarterial chemoembolization
TRAEs: Treatment-related adverse events

## 1 Introduction

Hepatocellular carcinoma (HCC) is characterized by an insidious onset and limited detectability at early stages, with approximately 70% of patients diagnosed at intermediate or advanced disease stages. Consequently, a substantial proportion of patients are no longer candidates for curative surgical resection and must rely on non-surgical locoregional therapies and systemic antitumor treatments.^1^ Hepatectomy and local ablation remain the principal curative approaches; however, postoperative recurrence continues to represent a major clinical challenge. The 5-year recurrence rate following curative hepatectomy approaches 70%, significantly limiting long-term survival outcomes.^2^^,^^3^ Moreover, even among patients with recurrent HCC who meet the Milan criteria, the 5-year recurrence rate after repeat curative resection remains similarly high.^4^, ^5^, ^6^ These findings reveal the critical importance of effective adjuvant strategies to reduce recurrence and improve overall survival (OS) after curative hepatectomy.^7^^,^^8^

A variety of adjuvant treatment strategies for HCC have been reported to date; however, the recommendations regarding adjuvant therapy remain inconsistent across international HCC guidelines. Although adoptive immunotherapy has shown modest benefit, its implementation is complex and thus is not routinely applied in clinical practice.^9^^,^^10^ By contrast, antiviral therapy for hepatitis B virus (HBV)-related HCC has achieved broad consensus, with multiple studies confirming both short- and long-term clinical benefits.^11^^,^^12^ Postoperative adjuvant therapy with PD-1 inhibitors has shown potential to improve recurrence-free survival (RFS) in selected patient subgroups,^13^, ^14^, ^15^, ^16^ but large-scale randomized controlled trials (RCTs) are still necessary to further confirm its efficacy, safety, and optimal patient selection.

The Chinese HCC guidelines additionally recommend adjuvant approaches including antiviral therapy, Huaier granules, and transarterial chemoembolization (TACE).^17^ Accordingly, many liver surgery centers in China have adopted 1–3 cycles of postoperative TACE in patients with high-risk recurrence features.^18^^,^^19^ By contrast, major international guidelines, including those from the American Association for the Study of Liver Diseases,^20^ European Association for the Study of the Liver,^21^ European Society for Medical Oncology,^22^ Barcelona Clinic Liver Cancer group,^23^ British Society of Gastroenterology,^24^ Asian Pacific Association for the Study of the Liver,^25^ and national guidelines from Republic of Korea,^26^ Japan,^27^ and Republic of India,^28^ do not recommend routine adjuvant TACE following curative resection.

The Chinese Liver Cancer Staging (CNLC)^29^ guideline strongly recommends adjuvant TACE after curative hepatectomy, largely based on two RCTs published in 2018.^30^^,^^31^ However, these studies did not specify whether patients with tumor staining on hepatic arteriography were excluded.^30^^,^^31^ Similarly, a recent retrospective analysis reported improved RFS and OS with postoperative TACE in selected patients with intermediate-to advanced-stage HCC and microvascular invasion, although patients with intraparenchymal tumor staining during TACE were not excluded.^32^ Advances in imaging further complicate interpretation of these findings. Historically, many patients underwent preoperative contrast-enhanced computed tomography (CT) without magnetic resonance imaging (MRI), whereas contrast-enhanced MRI now enables improved detection of intrahepatic microlesions, facilitating more complete resection. Notably, a recent RCT in patients with American Joint Committee on Cancer TNM stage I/II HCC demonstrated no RFS benefit with adjuvant TACE after curative hepatectomy.^33^

The TACE procedure begins with digital subtraction angiography (DSA), which can identify residual intrahepatic lesions through tumor staining that were not detected on preoperative imaging. The presence of tumor staining indicates that the criteria for standard curative hepatectomy may not have been fully met. However, prior studies^30^^,^^31^ have not accounted for this factor in their inclusion criteria, potentially contributing to the reported efficacy of adjuvant TACE. To further investigate this issue, we reviewed adjuvant TACE data from our center. Among 1390 patients who underwent curative hepatectomy between June 2019 and December 2023, 433 (31.1%) received adjuvant TACE 4–8 weeks postoperatively. Of these, 118 patients (27.3%) exhibited intrahepatic tumor staining on DSA, and these were the cases that actually benefited from postoperative TACE.^34^ These findings suggest that the apparent benefit of adjuvant TACE may primarily arise from detection of residual disease via DSA, rather than from the intrinsic therapeutic effects of embolization or chemotherapeutic agents.

**Fig. 1:**
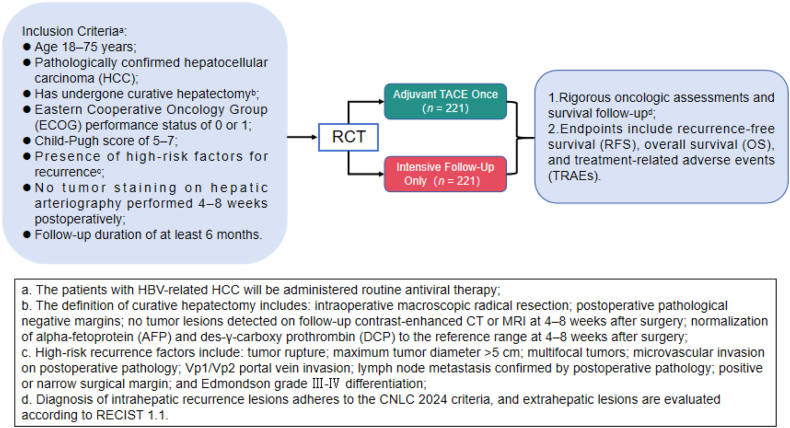
Overview of the study design and participant flow.

In this context, this study is designed as a rigorously conducted multicenter phase III RCT that emphasizes truly curative resection and excludes patients with intraoperative tumor staining detected by DSA. The trial aims to evaluate the effect of adjuvant TACE on RFS in HCC patients with high-risk recurrence factors (Fig. 1). The findings of this study are expected to clarify the clinical value of adjuvant TACE, provide high-level evidence for precision treatment strategies and optimize resource utilization in HCC management.

## 2 Methods

### 2.1 Objectives

This study is a multicenter phase III RCT, aims to rigorously evaluate the clinical efficacy of adjuvant TACE of HCC patients with high-risk recurrence factors following curative hepatectomy. The primary endpoint of this study is to investigate whether adjuvant TACE could improve RFS in high-risk HCC patients following curative hepatectomy. The key secondary endpoints include the safety and tolerability of adjuvant TACE—such as incidence of treatment-related adverse events (TRAEs) and serious adverse events (SAEs), and the rate of treatment discontinuation attributable to TRAEs or SAEs—as well as the effects of adjuvant TACE versus intensive follow-up on OS, median RFS, and time to tumor recurrence.

RFS is defined as the interval from randomization to the first imaging-confirmed recurrence of HCC—whether intrahepatic or extrahepatic—or death from any cause. TRAEs is defined as any unfavorable or unintended medical occurrences following informed consent, irrespective of causality. SAEs is defined as events that result in death, are life-threatening, require hospitalization or prolong hospitalization, result in persistent or significant disability, cause congenital anomalies, or represent other medically significant conditions. OS is defined as the time from randomization to death from any cause; time to recurrence, defined as the interval from curative hepatectomy to first imaging-confirmed recurrence.

### 2.2 Study setting

This study will be conducted across more than ten hospitals in Guangxi, China, a region with a high incidence of HCC.

### 2.3 Coordinating center and trial governance

This trial has received ethical approval from the Ethics Committee of Guangxi Medical University Cancer Hospital (Approval No. CS2025 (115)) and additional participating centers, including the Ethics Committee of the Second Affiliated Hospital of Guangxi Medical University (Approval No. 2025-KYC (0562)), the Ethics Committee of Liuzhou People's Hospital (Approval No. KY2026-030-01) and the Ethics Committee of First People's Hospital of Nanning (Approval No. YJ2025-019). The study is conducted in accordance with the principles of the Declaration of Helsinki, Good Clinical Practice (GCP) guidelines, and all applicable Chinese laws and regulations governing pharmaceutical research and data protection.

### 2.4 Recruitment

Eligible patients will be consecutively enrolled from participating centers. All participants will receive comprehensive information regarding the potential benefits and risks of the study interventions to support informed participation and sustained recruitment.

### 2.5 Informed consent

Written informed consent will be obtained from each participant or their legally authorized representative prior to initiation of any study-specific procedures. Participants will be fully informed of the study objectives, procedures, potential risks and benefits, alternative treatment options, data confidentiality measures, and their right to withdraw at any time. Adequate time will be provided for questions and consideration. The investigator or designated study personnel will document consent by signature and date, retain the original signed form at the study site, and provide a copy to the participant.

### 2.6 Patients

Following hepatic angiography confirming the absence of intrahepatic tumor staining, eligible participants will be randomly assigned in a 1:1 ratio to either the adjuvant TACE group or the intensive follow-up group. In the adjuvant TACE group, participants will undergo a single session of TACE after confirmation of no tumor staining on hepatic arteriography, followed by standardized intensive follow-up. Participants allocated to the intensive follow-up group will receive routine surveillance only after hepatic angiography. Patients in both groups were followed until death or study termination. Key landmarks defining follow-up cessation included: disease progression (defined as HCC recurrence or metastasis), death, withdrawal of informed consent, initiation of a new antitumor therapy, or any other protocol-specified reason for treatment discontinuation.

#### 2.6.1 Eligibility criteria

Eligible participants must meet all of the following criteria: age 18–75 years; Eastern Cooperative Oncology Group performance status (ECOG PS) of 0–1; Child–Pugh class A or B7 (score 5–7); prior radical hepatic resection at a participating center; and histopathological confirmation of HCC. All participants must undergo hepatic angiography 4–8 weeks postoperatively, demonstrating no intrahepatic tumor staining. Additional requirements include no prior systemic antitumor therapy for HCC, adequate organ and bone marrow function, and an estimated life expectancy greater than 6 months. Participants must present with at least one high-risk recurrence factor, including tumor rupture, maximum tumor diameter > 5 cm, multifocal disease, microvascular invasion on postoperative pathology, Vp1/Vp2 portal vein invasion, lymph node metastasis confirmed by postoperative pathology, positive or narrow surgical margins, or Edmondson grade III–IV differentiation. Participants must be capable of understanding study procedures and providing written informed consent. Women of childbearing potential must confirm absence of pregnancy and, where applicable, all participants must use highly effective contraception (annual failure rate < 1%) for at least 120 days following hepatic angiography.

#### 2.6.2 Exclusion criteria

Participants will be excluded if they lack pathological confirmation of HCC or have a history of other malignancies within 5 years prior to enrollment. Additional exclusion criteria include a history of hepatic encephalopathy, liver transplantation, or clinically significant pleural effusion, ascites, or pericardial effusion after curative hepatectomy. Participants with a history of drug allergy, active pulmonary tuberculosis, active syphilis infection, autoimmune disease, or long-term glucocorticoid use will also be excluded. Further exclusions include severe infection within 4 weeks prior to the first intervention or prior receipt of systemic antitumor therapy. Pregnant or lactating women are not eligible. Participants unable to adhere to the treatment protocol or complete follow-up will also be excluded.

#### 2.6.3 Criteria for discontinuation

Participants may discontinue study treatment or be withdrawn prematurely under the following conditions: initiation of antitumor therapy other than adjuvant TACE or antiviral therapy for hepatitis B or C after curative hepatectomy, including but not limited to anti-angiogenic tyrosine kinase inhibitors or immune checkpoint inhibitors; disease progression requiring modification of treatment strategy; occurrence of SAEs or intolerance to the study intervention (all SAEs and adverse events will remain included in safety analyses); withdrawal of consent by the participant; investigator-determined discontinuation for medical reasons; inadequate compliance with study requirements; confirmed pregnancy, which will also be reported as an SAE; use of prohibited concomitant medications or substances that may affect safety or efficacy assessments; development of a condition that substantially alters clinical status or study endpoints; diagnosis of a second primary malignancy; or death.

### 2.7 Strategies to improve adherence to interventions

The interventions employed align with established clinical practice. Participants in the adjuvant TACE group will receive treatment in accordance with CNLC recommendations,^29^ facilitating early detection of residual disease. Participants in the intensive follow-up group will avoid unnecessary treatment-related costs and potential adverse effects. The study will be conducted in accordance with GCP and applicable ethical standards. Investigators will maintain consistent and thorough communication with participants to promote adherence, reduce loss to follow-up, and minimize missing data. Prior to enrollment, investigators will provide a detailed explanation of study procedures, potential benefits, associated risks, and participant rights, including the right to withdraw consent at any time without consequence. Enrollment will proceed only after written informed consent has been obtained.

All analyses will follow the intention-to-treat principle. Participants who discontinue treatment or withdraw from the study prior to completion of the protocol-specified regimen will be encouraged to continue follow-up and complete all scheduled assessments. At the time of discontinuation or withdrawal, all end-of-treatment evaluations will be performed. For participants who withdraw for reasons other than disease progression, imaging assessments will remain mandatory.

### 2.8 Interventions

All enrolled participants will undergo hepatic arteriography 4–8 weeks following curative hepatectomy. With the patient in the supine position, standard aseptic preparation and sterile draping of the right groin will be performed. After local anesthesia with 5 mL of 2% lidocaine at the right femoral artery puncture site, a 5F arterial sheath will be inserted using the Seldinger technique. A 5F hepatic catheter will then be advanced into the celiac trunk for angiographic assessment. The hepatic arterial system, including the left and right hepatic artery branches, will be systematically evaluated to identify intrahepatic tumor staining. Detection of one or more foci of tumor staining will indicate that the initial hepatectomy did not meet criteria for curative resection, and such patients will be excluded. Participants without evidence of tumor staining will be randomly assigned to either the adjuvant TACE group or the intensive follow-up group.

In the adjuvant TACE group, superselective catheterization will be performed targeting the hepatic resection margin, followed by embolization using an emulsion of 50 mg lobaplatin and 3–5 mL ethiodized poppyseed oil. This regimen is based on prior RCTs demonstrating a reduction in postoperative HCC recurrence with adjuvant TACE.^30^^,^^31^ Polyvinyl alcohol embolic microspheres will not be utilized, as they are not part of the standard adjuvant protocol. In the intensive follow-up group, hepatic arteriography will be performed without administration of embolic or chemotherapeutic agents. Upon completion of the procedure, the catheter and arterial sheath will be removed, and manual compression will be applied to achieve hemostasis. Adjuvant TACE will be administered as a single session only.

### 2.9 Assignment of interventions: allocation

#### 2.9.1 Sequence generation

Randomization will be performed using computer-generated random number tables managed by Good Clinical Practice-compliant research centers at participating sites. Personnel responsible for sequence generation will have no involvement in study conduct. The interval between randomization and administration of adjuvant TACE will not exceed 1 week. During angiography, if tumor staining is identified in the remnant liver, TACE will be performed for clinical management; however, such participants will be excluded from the study.

#### 2.9.2 Concealment mechanism

Allocation materials will be securely stored in sealed envelopes. Both the envelopes and the original documentation will be retained by the departmental records management unit for auditing and verification purposes.

#### 2.9.3 Implementation

Following confirmation of eligibility, a designated study staff member will open the sealed envelope to obtain the assigned randomization number, which will determine allocation in a 1:1 ratio to either study group.

#### 2.9.4 Blinding

This study adopts a multiple blinding design. The doctor performing interventional operations will be aware of treatment allocation, whereas investigators, participants and data analysts will remain blinded throughout the study until unblinding.

#### 2.9.5 Unblinding procedures

Emergency unblinding may be conducted in the event of a medical emergency or upon request by regulatory authorities.

### 2.10 Concomitant medications during the study

All medications administered within 30 days prior to screening and all concomitant medications used during the study period will be documented in the electronic case report form.

Supportive treatments permitted during the study include antiviral therapy for hepatitis B virus or hepatitis C virus infection,^35^ antibiotics, analgesics, hormonal therapies, intravenous fluid support, psychological interventions, palliative surgical procedures, and other measures necessary for symptom management.

Participants are prohibited from initiating any medications without prior investigator approval in the absence of disease recurrence or metastasis. From study entry until 120 days after the final study intervention, the use of investigational agents, antitumor therapies, immunosuppressive drugs, and live vaccines is strictly prohibited.

### 2.11 Follow-up procedure

Participants allocated to the adjuvant TACE group will receive a single TACE procedure following hepatic angiography, followed by standardized intensive follow-up, whereas those in the intensive follow-up group will undergo surveillance alone after angiography. Follow-up assessments will be conducted every 3 months (±7 days) from the time of first intervention, and every 6 months (±7 days) after 2 years. At each visit, participants will undergo comprehensive evaluation, including contrast-enhanced CT or MRI of the chest and upper abdomen, measurement of tumor markers such as alpha-fetoprotein and abnormal prothrombin, and additional assessments as detailed in Table 1.

**Table 1:** Schedule of study assessments and study timeline.

Phase	Screening period	TACE treatment period	Safety Visit o	Survival Visit p
		C1/D1		
Days	−28∼-1	1	30 days Post-TACE	Every 90 days
Time Window (Days)	NA	+3	±7	±7
**General Study Process**				
Written Informed Consent^a^	×			
Inclusion/Exclusion Criteria	×			
Demographics/Medical History/Prior Treatment History b	×			
Previous Concomitant Medications	×	×	×	
Vital Signs^c^	×	×	×	
Weight/Height^d^	×	×	×	
Comprehensive Physical Examination	×	×	×	
Eastern Cooperative Oncology Group performance status	×	×	×	
12-lead Electrocardiogram (ECG) e	×	×		
**Laboratory Assessment**				
Complete Blood Count f	×	×	×	
Blood Biochemistry f	×	×	×	
Urinalysis f	×	×	×	
Coagulation Profile g	×	×	×	
Pregnancy Test h	×			
Thyroid Function Tests i	×	×	×	
Cardiac Enzyme Panel i	×	×	×	
Virological Antibody Testing j	×			
If HCV antibody is positive, test for HCV RNA k	×			
If HBsAg and/or HBcAb are positive, test for HBV-DNA l	×	×	×	
**Safety Monitoring and Survival**				
Adverse Event Assessment m	×	×	×	×
Subsequent Anti-tumor Treatment				×
Survival Status				×
**Efficacy Assessment**				
Tumor Imaging Assessment n	×		×	
**Investigational Drug Administration**				
TACE		×		

Notes.

a Written informed consent must be obtained prior to initiation of any protocol-specified procedures.

b Prior antitumor treatment history includes all therapies administered for HCC, such as chemotherapy, radiotherapy, and surgical interventions.

c Vital signs include body temperature, pulse rate, respiratory rate, and blood pressure.

d Height will be measured only during the Screening Period. Body weight will be recorded prior to each treatment administration. If body weight varies by less than 10% from baseline (defined as the day of first study treatment), baseline weight will be used for dose calculations; otherwise, the weight measured on the day of treatment will be used.

e A 12-lead ECG will be performed during the Screening Period, prior to each treatment cycle beginning from Cycle 2, and during the safety follow-up period.

f Complete blood count includes red blood cell count, hematocrit, hemoglobin, platelet count, white blood cell count, and differential counts (lymphocytes, monocytes, neutrophils, eosinophils, and basophils). Blood biochemistry includes liver function parameters (total bilirubin, direct bilirubin, aspartate aminotransferase, alanine aminotransferase, γ-glutamyl transferase, albumin, total protein, alkaline phosphatase, lactate dehydrogenase, and creatine kinase), renal function markers (urea and creatinine), blood electrolytes (sodium, potassium, chloride, magnesium, calcium, phosphorus), amylase, and blood glucose. Urinalysis includes pH, urine protein, red blood cells, white blood cells, glucose, and specific gravity. Participants with urine protein ≥2+ at screening will undergo 24-h urine protein quantification. These assessments will be performed within 7 days prior to first treatment, prior to each subsequent cycle (from Cycle 2 onward), and during safety follow-up. All tests will be conducted at study sites.

g Coagulation profile includes prothrombin time and international normalized ratio. Assessments will be performed within 7 days prior to first treatment, prior to subsequent treatment cycles (from Cycle 2 onward), and during safety follow-up at each study site.

h Female participants of childbearing potential will undergo serum pregnancy testing within 72 hours prior to the first treatment administration. If urine testing is inconclusive, serum testing will be used for confirmation. All tests will be conducted at study sites.

i Thyroid function tests and cardiac enzyme panels will be performed within 28 days prior to first treatment, prior to each treatment cycle (from Cycle 2 onward), and during safety follow-up at study sites.

j Virological testing includes human immunodeficiency virus, HCV antibody, and hepatitis B panel (HBsAg, HBsAb, HBcAb, HBeAg, HBeAb), performed within 28 days prior to first treatment during screening.

k For participants with positive HCV antibody, HCV-RNA viral load will be assessed every (21 ± 7) days from first treatment or earlier if clinically indicated.

l For participants with positive HBsAg and/or HBcAb, HBV-DNA viral load will be assessed every (21 ± 7) days from first treatment or earlier if clinically indicated.

m Adverse events and laboratory safety parameters will be evaluated according to Common Terminology Criteria for Adverse Events version 5.0. Definitions, grading, causality assessment, reporting timelines, and management procedures are specified in Section 8 of the protocol.

n Tumor assessments will be conducted in accordance with Response Evaluation Criteria in Solid Tumors version 1.1 (RECIST 1.1)^36^ and modified RECIST (mRECIST).^37^ Imaging evaluations will primarily consist of contrast-enhanced computed tomography (CT) or magnetic resonance imaging (MRI), with mandatory inclusion of thoracic and abdominal regions. At baseline, within 28 days prior to enrollment, additional imaging assessments—including pelvic CT or MRI, cranial MRI, and whole-body bone scan—are required. If cervical lymph node enlargement is present, contrast-enhanced neck CT should also be performed. Positron emission tomography/computed tomography (PET/CT) may be used as a baseline screening modality; if abnormalities are detected, corresponding contrast-enhanced CT or MRI examinations must be performed to facilitate subsequent longitudinal comparisons. For participants without evidence of metastatic disease at baseline, routine cranial, pelvic, and whole-body bone imaging is not required during follow-up unless clinically indicated. Imaging modalities and techniques should remain consistent throughout the study for each participant. Tumor imaging evaluations will be performed every 6 weeks (±7 days) starting from the first administration of investigational treatment, and subsequently every 12 weeks (±7 days) after 54 weeks. Upon the first radiological assessment indicating progressive disease based on RECIST 1.1, participants with clinically stable disease, absence of rapid radiological progression, and investigator-determined potential for continued clinical benefit may continue the current investigational treatment. In such cases, a confirmatory imaging assessment must be performed after a minimum interval of 4 weeks in accordance with RECIST 1.1. If progression is confirmed, the participant must discontinue investigational treatment; if not confirmed, treatment may continue with ongoing imaging evaluations per the protocol-defined schedule until radiologically confirmed progression. For participants who discontinue investigational treatment for reasons other than objective disease progression, tumor imaging assessments must be conducted at the time of treatment discontinuation and continued thereafter according to the protocol-specified schedule until the earliest occurrence of initiation of new antitumor therapy, confirmed disease progression, loss to follow-up, death, or withdrawal of informed consent. Unscheduled imaging assessments may be performed at any time if clinically indicated. In cases of clinically unstable disease following initial radiological progression, confirmatory imaging after 4–6 weeks is not required, and investigational treatment should be discontinued immediately.

o Safety follow-up will be conducted at (30 ± 7) days after TACE treatment or prior to the initiation of any new antitumor therapy, whichever occurs first. All adverse events (AEs) that occur prior to the safety follow-up visit must be documented and followed until resolution to Grade 0–1 return to baseline status, or until the investigator determines that further follow-up is not clinically warranted based on appropriate justification (e.g., no reasonable expectation of recovery or clear clinical improvement), whichever occurs first. Serious adverse events (SAEs) occurring within 90 days after study treatment administration or prior to the initiation of subsequent antitumor therapy (whichever occurs first) must be continuously monitored and documented in detail until resolution, stabilization, or return to baseline clinical status, in accordance with protocol-defined safety reporting procedures.

p Survival follow-up will be conducted every 90 days (±7 days) after completion of the safety follow-up visit. These follow-up assessments may be performed via in-person visits or telephone contact, as appropriate, to collect survival status and relevant clinical information.

The participant timeline is illustrated in Fig. 1.

### 2.12 Sample size calculation

Sample size estimation was performed using PASS 2021 (version 21.0.3) for a noninferiority randomized controlled trial. Based on historical data from our center, the median RFS for patients with high-risk recurrence factors managed with intensive follow-up alone was 16.1 months,^13^ corresponding to a hazard rate of 0.043 (ln(2)/16.1). Enrollment is planned over 1 year, followed by at least 2 years of follow-up. With a one-sided α of 0.025, 80% power, and a 1:1 allocation ratio, 192 participants per group (total *n* = 384) are required to demonstrate noninferiority at a hazard ratio margin of 1.4, with an estimated 139 events per group. Assuming an annual attrition rate of 5%, an additional 58 participants are required, yielding a total sample size of 442.

Sensitivity analysis was conducted using an alternative median RFS of 19.0 months derived from a cohort excluding DSA-positive patients,^34^ corresponding to a hazard rate of 0.0365 (ln(2)/19.0). Under similar assumptions, 360 and 361 participants would be required in the experimental and control groups, respectively (total *n* = 721), to achieve 80% power at the same noninferiority margin. Given the practical constraints of an investigator-initiated study without commercial funding, enrollment of more than 700 participants is not feasible; therefore, a target sample size of at least 442 participants has been selected to ensure study reliability.

### 2.13 Statistical methods

All statistical analyses will be performed using SAS software (version 9.2). A one-sided superiority testing framework will be applied, with a significance level (α) of 0.05. Between-group comparisons will be reported with corresponding *p*-values and 95% confidence intervals. Continuous variables will be summarized as mean ± standard deviation for approximately normally distributed data, or as medians otherwise. Categorical variables will be presented as frequencies and percentages. Survival outcomes, including median RFS and OS, will be estimated using the Kaplan–Meier method with corresponding 95% confidence intervals. Group comparisons will be performed using the log-rank test, and survival curves will be generated accordingly.

### 2.14 Additional analyses

Predefined subgroup analyses of RFS will be conducted according to clinically relevant prognostic factors, including age, ECOG PS, tumor stage, tumor rupture, maximum tumor diameter, tumor multiplicity, microvascular invasion, Vp1/Vp2 portal vein invasion, lymph node metastasis, surgical margin status, and histologic differentiation grade.

### 2.15 Data collection and management

#### 2.15.1 Outcome assessment and data collection

All outcome measures and their scheduled time points are summarized in Table 1. The sponsor will establish and maintain a comprehensive quality assurance and quality control system in accordance with predefined standard operating procedures. This system will ensure that all aspects of trial conduct, including data collection and outcome assessment, comply with the study protocol, GCP guidelines, and applicable regulatory requirements.

#### 2.15.2 Data management

Case report forms (CRFs) will be completed for all enrolled participants by study investigators. Completed CRFs will undergo review by a clinical research assistant and subsequent verification and signature by the investigator, after which the data will be rendered non-modifiable. To ensure data accuracy and integrity, two independent investigators will perform double data entry into a centralized database. Following data verification and validation, the database will be formally locked to prevent further modification. Statistical analyses will be conducted by designated data analysts, and the results will be provided to investigators for preparation of the final study report. The central database will be protected against unauthorized access. All essential documents, including completed CRFs, signed informed consent forms, randomization envelopes, the study protocol, and any amendments, will be securely archived at investigational sites for a minimum of 5 years after study completion.

#### 2.15.3 Confidentiality

All study-related documents, including informed consent forms, CRFs, and allocation records, will be stored in secure, access-controlled facilities accessible only to authorized personnel.

#### 2.15.4 Management of biological specimens

No biological specimens will be collected for molecular or genetic analyses in this study.

#### 2.15.5 Data sharing statement

The full study protocol, participant-level dataset, and statistical code will be made publicly available following publication of the full study results.

### 2.16 Interim analyses

An initial interim analysis will be conducted after approximately 50 participants have undergone DSA, of whom an estimated 25 will have received TACE. Thereafter, safety data will be periodically reviewed by the data monitoring committee each time approximately 100 participants complete DSA, including approximately 50 participants in the TACE group. An interim analysis of RFS and a reassessment of sample size will be performed when approximately 50% of participants (*n* = 221) have been enrolled and have completed at least 6 months of follow-up or have withdrawn prematurely. A second interim analysis will be conducted one year after all participants have completed the study or upon early termination.

### 2.17 Oversight and monitoring

#### 2.17.1 Data monitoring committee

An Independent Data Monitoring Committee has been established under the GCP Office of Guangxi Medical University Cancer Hospital. Personnel from the GCP Office will maintain ongoing communication with study investigators to monitor trial progress and address operational issues. CRFs will be systematically reviewed to assess protocol adherence, data accuracy, completeness, and compliance with scheduled study visits. Upon participant enrollment, investigators are required to submit signed informed consent forms to the GCP Office for verification. In instances of data discrepancies or missing information, investigators will be required to complete data clarification forms to ensure data integrity. Any significant issues identified during monitoring, including serious violations of GCP standards or study protocol, will be reported to the Trial Management Team and relevant regulatory authorities. Investigators will be subject to auditing, monitoring, ethics review, and regulatory inspection, and must provide access to original study data as required.

#### 2.17.2 Adverse event reporting

Adverse events (AEs) will be graded according to the Common Terminology Criteria for Adverse Events (version 5.0). All AEs and SAEs will be comprehensively documented, including event type, onset and resolution dates, severity, relationship to the study intervention, and concomitant treatments. In addition to institutional reporting requirements, investigators must notify the principal investigator and the institutional ethics committee within 24 hours of becoming aware of any SAE. Participants will be followed until resolution of the event or return to baseline clinical status.

#### 2.17.3 Audit of trial conduct

The Trial Management Group will conduct annual audits to ensure compliance with the study protocol, regulatory standards, and GCP requirements.

#### 2.17.4 Protocol amendments

Any protocol modifications will be jointly reviewed and agreed upon by the sponsor and investigators. All amendments will be formally documented as protocol addenda and submitted to the relevant ethics committees for approval or notification in accordance with institutional policies.

### 2.18 Dissemination policy

This study has been registered at ClinicalTrials.gov (NCT07417397). Upon completion, anonymized individual-level data will be made available through a public repository (researchdata.org.cn). Study findings will be disseminated through presentations at scientific conferences and publication in peer-reviewed journals. Requests for access to unpublished data may be directed to the corresponding authors and will be considered in accordance with applicable ethical and confidentiality requirements.

## 3 Discussion

Hepatic resection and local ablation remain the primary curative treatment modalities for HCC; however, long-term outcomes are substantially limited by high recurrence rates, with approximately 70% of patients experiencing recurrence within 5 years after resection.^2^^,^^3^ The role of adjuvant therapy in this setting remains highly controversial. Notably, the IMbrave050 trial reported limited benefit of adjuvant immune checkpoint inhibition, leading most international guidelines to recommend intensive follow-up rather than routine postoperative therapy.^20^ By contrast, the CNLC strongly endorses TACE as an adjuvant strategy following curative hepatectomy,^17^^,^^29^ primarily based on two RCTs published in 2018.^30^^,^^31^ However, critical methodological limitations are evident in these studies, particularly the absence of explicit exclusion of patients with tumor staining identified on hepatic arteriography, which may reflect residual intrahepatic disease and thus incomplete, non-curative resection. A more recent RCT in patients with American Joint Committee on Cancer TNM stage I/II HCC demonstrated that adjuvant TACE does not improve RFS in those who have undergone true curative resection.^33^ These observations support the hypothesis that the apparent benefit of adjuvant TACE reported in earlier studies may be attributable to the treatment of residual lesions following non-radical resection, rather than to the intrinsic pharmacologic or embolic effects of TACE. Accordingly, when curative standards are achieved, defined by the absence of tumor staining on DSA, adjuvant TACE may confer no additional therapeutic advantage.

To address these unresolved issues, the present study was designed as a rigorously conducted multicenter phase III RCT that prioritizes confirmation of curative resection and excludes patients with tumor staining. This trial evaluates the impact of adjuvant TACE on RFS in patients with HCC who have high-risk recurrence factors following curative hepatectomy. A total of 442 participants will be enrolled, exceeding the sample sizes of prior studies^30^^,^^31^^,^^33^ to generate more robust and definitive evidence regarding the potential survival benefit of adjuvant TACE in this population. Overall, this trial is intended to provide high-level evidence to inform precision management strategies for HCC, support refinement of clinical guidelines, and promote the efficient allocation of healthcare resources.

## CRediT authorship contribution statement

**Chao-Man Huang:** Resources, Formal analysis, Investigation, Writing – review & editing. **Shu-Chang Chen:** Resources, Investigation. **Yi-He Yan:** Resources, Investigation. **Chuang Qin:** Resources, Investigation. **Shu-Qun Li:** Resources, Investigation. **Ze Su:** Resources, Investigation. **Xiao-Feng Dong:** Resources, Investigation. **Teng-Meng Zhong:** Resources, Investigation. **Fan-Jian Zeng:** Resources, Investigation. **Hong-Bing Yao:** Resources, Investigation. **Shao-Ping Liu:** Resources, Investigation. **Pei-Sheng Wu:** Resources, Investigation. **Ning Peng:** Resources, Investigation. **Jie Liu:** Resources, Investigation. **Liang Ma:** Resources, Investigation, Writing – review & editing. **Jian-Hong Zhong:** Writing – review & editing, Writing – original draft, Supervision, Project administration, Conceptualization, Data curation, Formal analysis, Funding acquisition, Investigation, Methodology, Resources, Visualization.

## Informed consent

All the subjects will be informed and sign a written consent to participate in this study.

## Availability of data and materials

The principal investigator, Jian-Hong Zhong, MD, PhD, will have access to the final trial dataset. Any data required to support the protocol can be supplied on request.

## Ethics statement

This study will adhere to the Helsinki Declaration as revised in 2000 and to institutional and national regulations in China. This trial protocol was reviewed and approved by the Ethics Committee of Guangxi Medical University Cancer Hospital (Approval No. CS2025(115)), the Ethics Committee of the Second Affiliated Hospital of Guangxi Medical University (Approval No. 2025-KYC (0562)), the Ethics Committee of Liuzhou People's Hospital (Approval No. KY2026-030-01) and the Ethics Committee of First People's Hospital of Nanning (Approval No. YJ2025-019).

## Organ donation

Not applicable.

## Data availability statement

Not applicable.

## Consent for publication

Not applicable—no identifying images or other personal or clinical details of participants are presented here or will be presented in reports of the trial results. The participant information materials and informed consent form are available from the corresponding author on request. When signing the informed consent, all participants will be informed that the relevant data will be compiled and published after the end of the study.

## Animal treatment

Not applicable.

## Declaration of generative AI and AI-assisted technologies in the writing process

Not applicable.

## Funding

This work was supported by the Guangxi key research and development plan (GuiKe AB25069099 and GuiKe AB24010082), the Clinical Discipline Construction Project of Guangxi Medical University (GXMULJZ202403), and the First-class discipline innovation-driven talent program of Guangxi Medical University. The funders have no role in the design and conduct of the present study; collection, analysis, and interpretation of the data; or writing, review, or approval of the manuscript. The above funds are not directly related to this trial. However, these fundings relate to a wider group of projects and apply to this study.

## Declaration of competing interest

The authors declare that they have no known competing financial interests or personal relationships that could have appeared to influence the work reported in this paper.
